# Ab Initio Insights into Zinc Dialkyldithiophosphate Linkage Isomers and Oxidative Degradation: Implications for Tribology

**DOI:** 10.1021/acsanm.5c01971

**Published:** 2025-07-09

**Authors:** Francesca Benini, Paolo Restuccia, Enrico Pedretti, Maria Clelia Righi

**Affiliations:** Department of Physics and Astronomy, 9296 University of Bologna, Viale Berti Pichat 6/2, Bologna 40127, Italy

**Keywords:** ZDDP, DFT, friction, tribofilm, lubricant additive, antiwear

## Abstract

Zinc dialkyldithiophosphates (ZDDPs) are widely used as antiwear additives in lubricants, forming protective tribofilms that reduce wear and friction in metallic contacts. However, the atomic-scale mechanisms governing their performance remain poorly understood. A key factor is the role of linkage isomers—alternative molecular forms in which sulfur (S) and oxygen (O) atoms exchange positions within the ligand structure. These isomers arise through alkyl group transfers and may significantly influence tribofilm formation. Using density functional theory (DFT), we systematically characterize ZDDP linkage isomers by analyzing their stability, vibrational spectra, and dissociation pathways. Our results show that linkage isomers are more stable than standard ZDDP forms due to the greater strength of Zn–O bonds and primarily dissociate through Zn–S­(O) bond cleavage under a 1 GPa load at ferrous interfaces. Additionally, we explore oxidative degradation pathways, where S atoms are replaced by O, altering the molecular stoichiometry. We find that oxidation is favorable in the gas phase, and it is exothermic when mediated by a ferrous substrate. To support experimental validation, we provide vibrational spectra for these isomers, enabling direct comparison with spectroscopic measurements. Bond strength analysis via static fragmentation and Integrated Crystal Orbital Overlap Population (ICOOP) further elucidates their structural stability.

## Graphical abstract

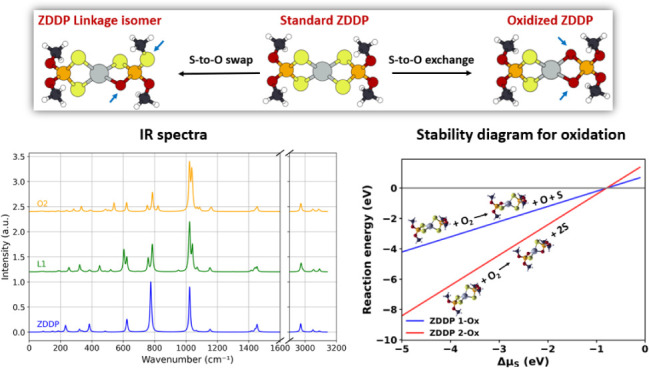

## Introduction

Frictional losses in mechanical systems pose a significant challenge, accounting for considerable energy inefficiencies and environmental impact. For instance, it is estimated that approximately 21.5% of the energy extracted from fuel in passenger cars is actually employed to move the car, with approximately one-third of this energy lost solely to frictional dissipation.^1^ Antiwear additives such as zinc dialkyldithiophosphates (ZDDPs) are crucial in reducing wear and friction in metallic sliding components, especially under boundary lubrication conditions. ZDDPs, widely used since their introduction in the 1940s, exhibit remarkable antiwear properties through the formation of protective films, including thermal films formed at elevated temperatures and tribofilms generated under mechanical stress.^2^ The antiwear efficacy of ZDDPs is attributed to their complex decomposition and tribochemical transformation pathways. Extensive experimental efforts have provided a wealth of information regarding the chemical composition and morphology of the ZDDP tribofilm.^3–6^ This film, usually measuring between 80 and 150 nm in thickness and consisting of flat-topped pads,^6^ features a superficial layer composed of zinc and iron polyphosphates, which act as barriers to surface wear and corrosion. Beneath this layer, closer to the steel surface, there is a sulfur-rich layer.^4^

The exact process leading to tribofilm formation is still widely debated in the literature and, in general, unclear. The dissociation of ZDDP molecules is suggested to involve the formation of transient intermediates, referred to as linkage isomers, which result from alkyl group transfers between oxygen and sulfur atoms.^7,8^ These isomers might facilitate subsequent reactions leading to the generation of phosphorus- and sulfur-containing species, which polymerize to form zinc thiophosphate networks.^9–14^ However, while these isomers are hypothesized to play a crucial role in both thermal and tribochemical film formation, their formation, stability, and reactivity in different environments are not yet fully understood. These gaps hinder the development of a unified understanding of ZDDP antiwear capabilities, limiting progress in designing environmentally safer alternatives with comparable performance. The presence of oxygen in the working environment plays a critical role in the formation and characteristics of ZDDP-derived tribofilms. Several studies suggested that the presence of dissolved oxygen in lubricants, as well as provided by an oxidized surface, affects tribofilm formation.^15–18^ Specifically, recent comparative experimental tribological studies conducted in ambient air versus inert atmospheres such as argon have demonstrated that oxygen significantly enhances tribofilm growth.^19^ In oxygen-rich environments, tribofilms form more rapidly and exhibit a higher degree of depolymerization, resulting in shorter-chain polyphosphates. These shorter chains tend to produce more compact and durable films. Conversely, tribofilms formed in argon atmospheres tend to contain longer polyphosphate chains and higher sulfur content, suggesting limited oxidation and possible formation of polythiophosphate structures. This is attributed to the lack of available oxygen, which inhibits the typical S-to-O exchange and restricts the conversion of soft long-chain polyphosphates into more stable short-chain variants.^15–19^ Computational simulations provide valuable insight into the atomic-scale mechanisms governing lubrication and wear. Among these, third-order density-functional tight-binding (DFTB3) methods have been effectively applied to study the influence of contact pressure and shear stress on the decomposition of ZDDP and the formation of tribofilms on diamond-like carbon (DLC) surfaces.^20^ Density Functional Theory (DFT) also serves as a widely used tool in computational tribochemistry, supporting investigations into diverse tribological phenomena, including the behavior of lubricant additives,^21,22^ interfacial adhesion,^23^ and, more recently, triboelectric effects.^24^ Studies employing DFT and ab initio molecular dynamics (AIMD) have demonstrated the ability to capture the finite-temperature behavior of ZDDP molecules and identify key decomposition pathways. For instance, AIMD has revealed distinct mechanisms for alkyl group transfer, olefin elimination, and thiophosphate polymerization, complementing experimental observations.^25,26^ Notably, computational studies have shed light on the distinct behavior of ZDDP isomers compared to the parent molecule, especially with respect to bond dissociation and their potential to form high-quality tribofilms. For example, one study has shown that ZDDP monomers and linkage isomers exhibit similar stability, with the latter species undergoing dissociation of Zn–S bonds and facilitating the loss of radicals, olefins, and sulfides.^25^ Furthermore, the dissociation of Zn–O bonds in isomers has been linked to an increase in electron-donating abilities, which enhances their potential to participate in reactions leading to antiwear film formation. These findings support the notion that isomerization and the dissociation of Zn–O bonds are crucial steps in the formation of zinc thiophosphate networks, a key component of antiwear films.^25,26^

In this study, we utilize DFT-based static calculations to explore how molecular modifications of ZDDP affect its properties. Specifically, we focus on characterizing linkage isomers by calculating their energetics and vibrational spectra. Additionally, we investigate the potential for oxidative degradation of ZDDP, as observed in MoDTC,^27,28^ leading to the formation of oxidized derivatives. These derivatives are then compared to the linkage isomers and the standard ZDDP molecule. We also examine the dissociation pathways of the investigated molecules when confined at ferrous interfaces under a typical 1 GPa load. To assess bond strength, we employ both static fragmentation and Crystal Orbital Overlap Population (COOP) analysis. Through a systematic analysis of these factors, we aim to uncover the fundamental role of isomerization in tribofilm formation. The insights gained from this study will deepen our understanding of ZDDP’s antiwear mechanisms and provide a foundation for the rational design of next-generation lubricants with reduced environmental impact.

## Computational Methods

The calculations in this study (except for IR and Raman spectra) have been performed using spin-polarized Density Functional Theory (DFT) as implemented in version 7.2 of the Quantum Espresso suite^29–31^ and Density Functional Perturbation Theory (DFPT) as implemented in version 6.4.2 of the VASP suite.^32^ To describe the exchange-correlation functional, we employed the generalized gradient approximation (GGA) with the Perdew–Burke–Ernzerhof (PBE) parametrization.^33^ For the Quantum Espresso calculations, we set 40 Ry as kinetic energy cutoff for the wave functions, while the cutoff for charge densities has been set to 320 Ry. To improve the description of occupations near the Fermi level, a Gaussian smearing with a width of 0.02 Ry was applied. The atomic electronic configuration was modeled using ultrasoft pseudopotentials based on the RRKJ parametrization.^34^ The structural geometries in all calculations were optimized using the standard convergence thresholds for energy and forces, set at 10^–4^ Ry and 10^–3^ Ry/Bohr, respectively. The geometric configuration and computational parameters for modeling the ZDDP molecule were previously validated by our research group.^35,36^ Specifically, the two lateral alkyl chains are simplified to methyl groups to reduce computational costs.

To obtain the infrared (IR) and Raman spectra of the investigated molecules, we employed VASP to compute the (zone-center) vibrational modes and the dipole and polarizability derivatives with respect to the normal modes.

The VASP calculations were performed with projector-augmented wave (PAW)^37^ pseudopotentials to represent the core electrons. The convergence threshold for the electronic self-consistent field (SCF) loop was set to the very low value of 10^–10^ eV to obtain well-converged forces. The Brillouin zone was sampled at the Γ point, the plane-wave basis set was truncated with a cutoff energy of 600 eV, and a Gaussian smearing of 0.01 eV was applied.

First, we optimized the geometries of the molecules with a strict convergence threshold on forces (5 × 10^–4^ eV/Å). Then we calculated the vibrational modes in the harmonic approximation with the DFPT implementation in VASP. The IR intensities of the normal modes were then obtained from the derivatives of the polarization with respect to atomic displacements, as in ref. 38, where we used the Born effective charges calculated with VASP^39,40^ for the polarization derivatives.

The Raman spectra were obtained within the Placzek approximation (valid for off-resonant eigenmodes) where the Raman intensity is proportional to the derivatives of the polarizability tensor with respect to atomic positions.^41^ These derivatives were calculated from the derivatives of the ion-clamped dielectric tensor $\epsilon_{ij}^{\infty }$ with respect to the atomic displacements, using a finite difference method, as in ref.^42^ For each mode, two structures were generated by displacing the atoms by ±δ = 1% along the eigenvector of the mode, and the dielectric tensor $\epsilon_{ij}^{\infty }\left(\pm \delta \right)$ was calculated for both displacement, again using the DFPT implementation in VASP. The Raman cross-section was calculated as in ref. 38, assuming the typically employed incident laser wavelength of 785 nm (to calculate the frequency of the scattered radiation) and a temperature of 300 K (for the Bose–Einstein occupancy factor).

Both IR and Raman spectra were obtained from the intensities of the modes as a superposition of Lorentzian peaks, broadened with a full width at half-maximum (fwhm) of 10 cm^–1^.

To identify the atoms that contributed the most to each vibrational mode, the displacements were visually inspected by generating animations with the *ase.vibrations* module of the ASE code.^43^

The dissociative pathway of ZDDP in tribological conditions was investigated by examining its behavior at the interface of two Fe surfaces, under an external load of 1 GPa. To simulate this scenario, uniaxial compressions were applied by decreasing the vertical dimension of the simulation cell, allowing the ZDDP molecule to interact with one side of the slab and its periodic replica, thereby mimicking the interface. This methodology facilitated the observation of molecular dissociation and provided insights into the bond-breaking processes occurring at the interface under load. The two modeled substrates consisted of clean (110) Fe surfaces and the same surface passivated with 0.25 ML of oxygen, to simulate the effect of oxidation which is typical in operating conditions. The efficacy of this model in simulating an hematite surface in terms of reactivity with molecules (specifically, with ZDDP) was previously assessed by our group.^36^ Specifically, for clean Fe we employed 6 × 8 orthorhombic supercells to eliminate unintended lateral interactions between periodic replicas. Each slab modeled the Fe (110) surface, comprising four atomic layers with 48 atoms per layer. Due to the size of the supercell, structural optimization was performed using Γ-point sampling only.

In order to investigate and compare the strengths of each bond, we evaluated the energy costs associated with different cuts of the molecule. To do so, we carried out static calculations at fixed ionic positions for isolated fragments in vacuum. The fragmentation energy *E*_frag_, representing the energy needed to dissociate each bond, was determined using the following expression:

1 $$E_{\mathrm{frag}}=\overset{N}{\underset{n=1}{\sum }}E_{f_{n}}-E_{\mathrm{mol}}$$

where, *N* represents the total number of fragments generated for each specific bond cleavage, $E_{f_{n}}$ denotes the computed energy of each fragment *n*, and *E*_mol_ is the total energy of the intact molecule in its isolated state. Additionally, we conducted bond order analysis and crystal orbital overlap population calculations using the LOBSTER software suite.^44–47^ Specifically, we analyzed the Integrated Crystal Orbital Overlap Population (ICOOP).

## Results and Discussion

### ZDDP Linkage Isomers

As an initial step in this study, we modeled the ZDDP molecule with methyl-terminated chains, focusing on the effects of isomerization while excluding the influence of substituents. This simplified model has been previously developed and validated by our group.^35,36^ Subsequently, we optimized the structures of all possible linkage isomers of the ZDDP molecule, considering configurations with one, two, three, and four S-to-O swaps, leading to alkyl chain transfer.

In this process, we identified five classes of isomers, labeled A, B, C, D, and E as shown in Figure 1. Among these, both classes B and C correspond to double S-to-O swaps but differ in their spatial arrangements: in class B, the two exchanges occur on the same side of the molecule, whereas in class C, they are on opposite sides. To quantify the relative stability of the isomers, we calculated the energy differences (ΔE) relative to the standard ZDDP molecule. These calculations yielded consistent ΔE values within each class of isomers, which can be attributed to the symmetry of the ZDDP molecule. The average ΔE values for each class are presented in Table 1.

**Figure 1:**
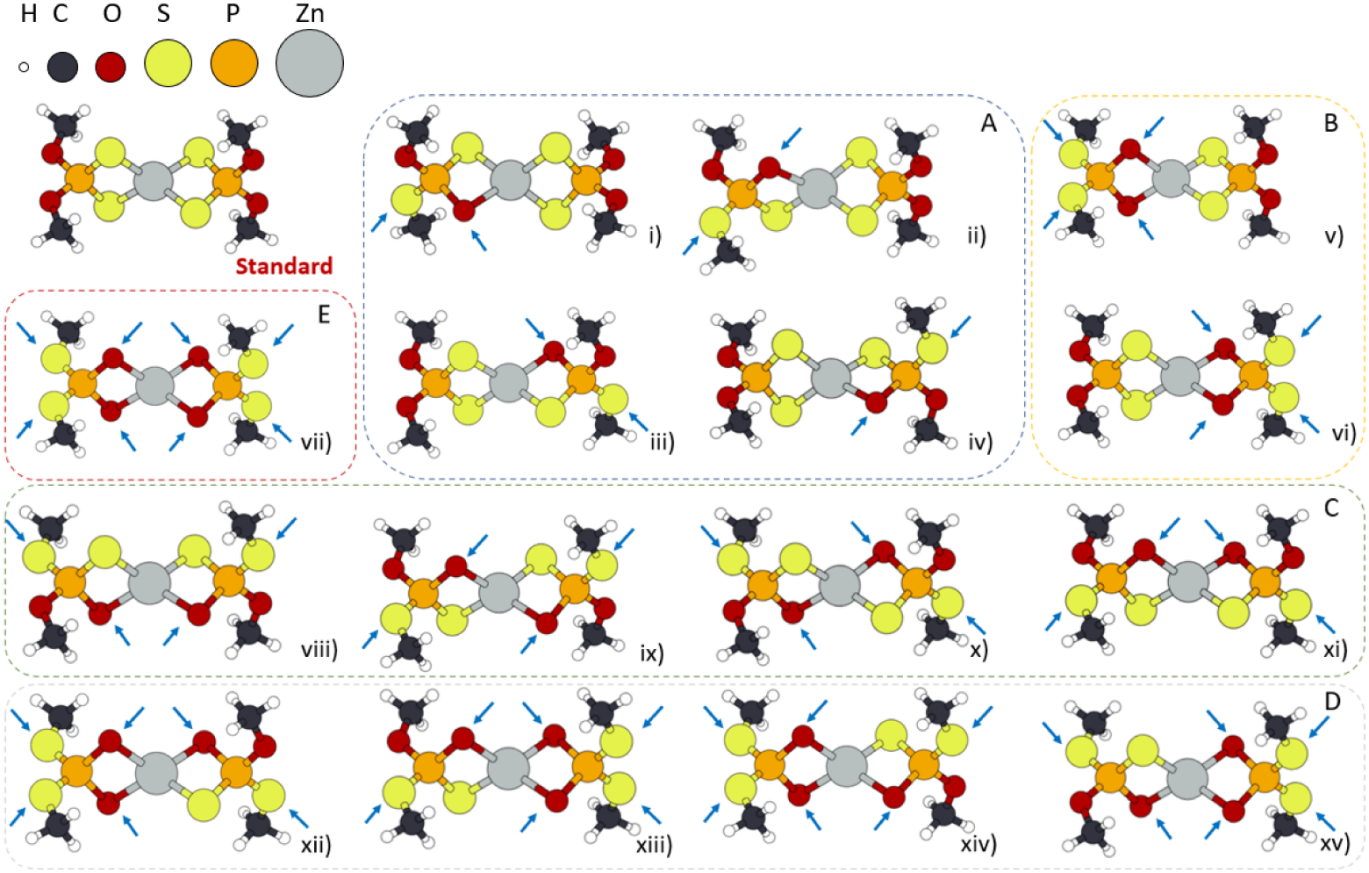
ZDDP isomers characterized by one (A), two (B, C), three (D), and four (E) S-to-O swaps. From now on, white atoms represent H, gray Zn, black C, yellow P, red O, and green S, respectively.

**Table 1:** Average Energy Difference between the Isomeric Form and the Standard ZDDP Molecule for Each Class of Isomers

Δ*E* (eV)				
A	B	C	D	E
–0.16	–0.18	–0.30	–0.32	–0.25

Following this, we selected two isomeric configurations for further study: isomer (iii) (class A) and isomer (vi) (class B) from Figure 1, which feature a single and double S-to-O swap, respectively. From this point onward, these isomers will be referred to as LI1-ZDDP and LI2-ZDDP, in line with existing literature.^25,26^

This selection is based on molecular symmetry. Given our previous energy analysis, we consider all other configurations as combinations of these two. Specifically, isomers in class A and B exhibit similar energy gains relative to the standard molecule (−0.16 eV and −0.18 eV, respectively), indicating that a single or double S-to-O swap involving the same P atom results in nearly the same stabilization. In contrast, isomers in class C and D, where two distinct P atoms undergo S-to-O swaps, show approximately double the energy gain (−0.30 eV and −0.32 eV). These configurations can be derived by combining the swaps in classes A and B, with their energy contributions summing accordingly. Notably, the negative ΔE values in Table 1 indicate that all the isomeric forms are energetically more favorable than the standard molecule. This can be attributed to the greater strength of Zn–O bonds compared to Zn–S bonds, as discussed in the “Bonds Strength Analysis: Static Fragmentation and ICOOP” Section. Specifically, in Cut 1 (Figure 5), the cleavage involves one Zn–O bond in LI1-ZDDP and two Zn–O bonds in LI2-ZDDP, increasing the energy required for this cut to 3.65 and 3.90 eV, respectively. In contrast, the standard ZDDP, where two Zn–S bonds are broken, requires only 3.57 eV, highlighting the stabilizing effect of Zn–O bonds.

### ZDDP Oxidative Degradation

XPS characterization of ZDDP-derived tribofilms formed under ambient conditions revealed an oxygen-to-phosphorus ratio substantially exceeding the 2:1 stoichiometry of the parent ZDDP molecule.^19,48^ This observation suggests that oxidative processes contribute significantly to tribofilm growth, with oxygen likely sourced either from dissolved molecular oxygen in the lubricant or via interaction with surface oxides.^15^ Experimental studies utilizing nuclear magnetic resonance (NMR) spectroscopy,^49^ alongside more recent investigations employing high-resolution mass spectrometry,^50^ have focused on elucidating the mechanisms underlying the thermal and oxidative stability of ZDDP. There is general consensus that oxidation in ZDDP occurs readily through intermolecular rearrangements, involving the exchange of sulfur, alkyl groups, phosphorus, and oxygen atoms, as well as the oxidation of P–S bonds to P–O bonds.^15,19,48–50^ To the best of our knowledge, a comprehensive stability diagram specifically addressing the oxidation behavior of ZDDP has not yet been reported in the literature. Similarly, experimental findings suggested that MoDTC, a common lubricant additive, undergoes oxidative degradation, changing its stoichiometry and increasing the oxygen content.^27^ Motivated by this, our group investigated oxygen-rich MoDTC in previous computational studies.^51,52^ In this work, we explore if a similar mechanism could occur for the ZDDP molecule, considering the uncertainty on the molecular-level processes involved in the tribofilm formation. We simulated two possible pathways for ZDDP oxidation: one involving O_2_ in the gas phase, and the other mediated by the substrate.

To evaluate the possibility for ZDDP to undergo oxidative degradation similarly to MoDTC, we realized a stability diagram (Figure 2). We analyzed oxidation reactions where sulfur atoms are replaced by oxygen, changing the molecule stoichiometry. This approach follows standard practices in surface science^53–57^ and allows us to examine the stability of oxidized ZDDP as a function of sulfur chemical potential.

**Figure 2:**
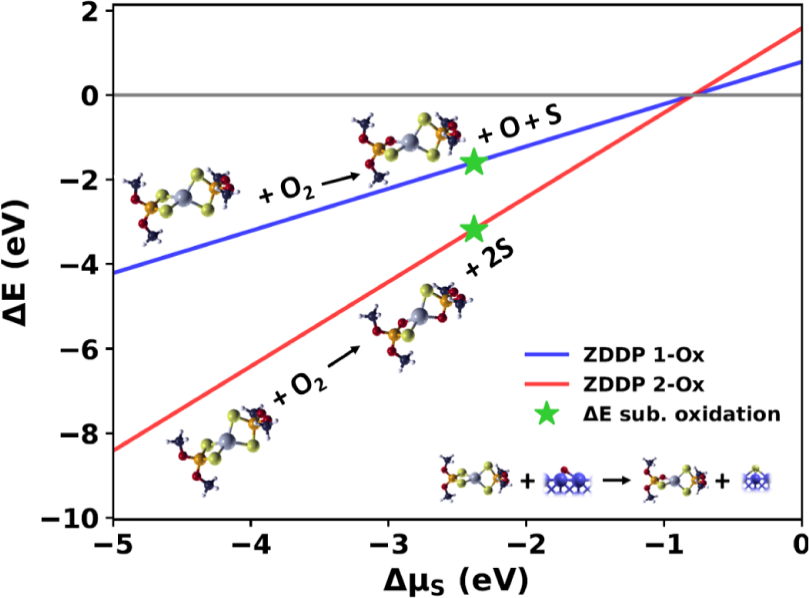
Stability diagram for the ZDDP oxidation processes. The blue (red) solid line represents oxidation with a single (double) S-to-O substitution involving an O_2_ molecule. The green stars indicate the reaction energy computed for substrate-mediated oxidation using eqs 8 and 9. A graphical representation for this chemical reaction path is shown in the inset.

For the O_2_-mediated pathway in the gas phase, we considered the following oxidation reactions:

2 $$\mathrm{ZDDP}+O_{2}\to \mathrm{O1‐ZDDP}+O+S$$

3 $$\mathrm{ZDDP}+O_{2}\to \mathrm{O2‐ZDDP}+2S$$

where O1-ZDDP and O2-ZDDP denote the molecule obtained with single and double S-to-O substitutions, respectively. Notably, these molecules differ from isomers in that their oxygen content is increased, whereas isomers maintain the same stoichiometry of the standard ZDDP molecule. The phase diagram in Figure 2 was built by computing the corresponding energy differences:

4 $$\Delta E=E_{O1-\mathrm{ZDDP}}+\mu_{S}+\mu_{O}-E_{\mathrm{ZDDP}}-E_{O_{2}}$$

5 $$\Delta E=E_{O2-\mathrm{ZDDP}}+2\mu_{S}-E_{\mathrm{ZDDP}}-E_{O_{2}}$$

where *E*_ZDDP_, *E*_O1‑ZDDP_, *E*_O2‑ZDDP_, and $E_{O_{2}}$ are the total energies of the corresponding molecular species after geometry optimization. The chemical potential of oxygen μ_O_ is fixed to half of the total energy of an O_2_ molecule, while the sulfur chemical potential (μ_S_) is varied between the energy of an S atom adsorbed on iron and the energy of an isolated S atom in vacuum, as performed in our previous study on MoDTC oxidation.^52^

It is evident that the ZDDP oxidation in the gas phase is energetically favorable over a wide range of S chemical potentials as the reaction energies are negative. Additionally, the double S-to-O substitution is consistently more favorable than the single one, suggesting that increased oxygen content further stabilizes ZDDP.

To better simulate real tribological conditions, we also considered substrate-mediated ZDDP oxidation, involving chemisorbed oxygen on an iron surface:

6 ZDDP+O‐on‐Fe→O1‐ZDDP+S‐on‐Fe

7 ZDDP+2O‐on‐Fe→O2‐ZDDP+2S‐on‐Fe

in which the oxidation occurs via atomic substitution mediated by a partially oxidized iron, where O atoms are chemisorbed on the Fe surface, as commonly observed under tribological conditions.^58^ We calculated the energy differences at 0.25 ML surface coverage as

8 $$\Delta E=E_{O1-\mathrm{ZDDP}}+E_{S_{\mathrm{ads}}}-E_{\mathrm{ZDDP}}-E_{O_{\mathrm{ads}}}$$

9 $$\Delta E=E_{O2-\mathrm{ZDDP}}+2E_{S_{\mathrm{ads}}}-E_{\mathrm{ZDDP}}-2E_{O_{\mathrm{ads}}}$$

in which $E_{S{\left(O\right)}_{\mathrm{ads}}}$ represent the energy of the adsorbed chemical species on a Fe surface. The results, shown as star markers in Figure 2, indicate a Δ*E* of −1.60 eV for the single substitution and −3.19 eV for the double one, confirming that substrate-mediated ZDDP oxidation is exothermic. These findings reveal that, similar to MoDTC, ZDDP could undergo oxidative degradation, which could influence tribofilm formation. The oxidation mechanism proposed for ZDDP may help explain the reduced sulfur content observed in tribofilms formed under oxygen-rich conditions, where sulfur is predominantly located near the iron substrate.^14^ In contrast, tribofilms generated in inert environments such as argon or nitrogen exhibit significantly higher sulfur concentrations throughout the film, as revealed by XPS depth profiling.^18,19^ This has been attributed to a limited oxygen/sulfur exchange during film formation, leading to the presence of sulfur-rich species like polythiophosphates.^19^ Such findings suggest that under oxidative conditions, sulfur is more readily displaced or incorporated into deeper interfacial regions. Future studies could explore how different ZDDP isomers impact tribofilm stability and friction reduction by identifying the optimal molecular composition that leads to low friction and wear.

### Calculated Vibrational Spectra

To facilitate experimental validation, we calculated and compared the vibrational spectra of the investigated molecules, as illustrated in Figure 3. The results provide insights into the molecular vibrations characteristic of each structure and the effects of specific modifications. The script we exploited enabled us to directly determine the correspondence between the atoms involved in the vibrational mode and peaks found in the spectra.

**Figure 3:**
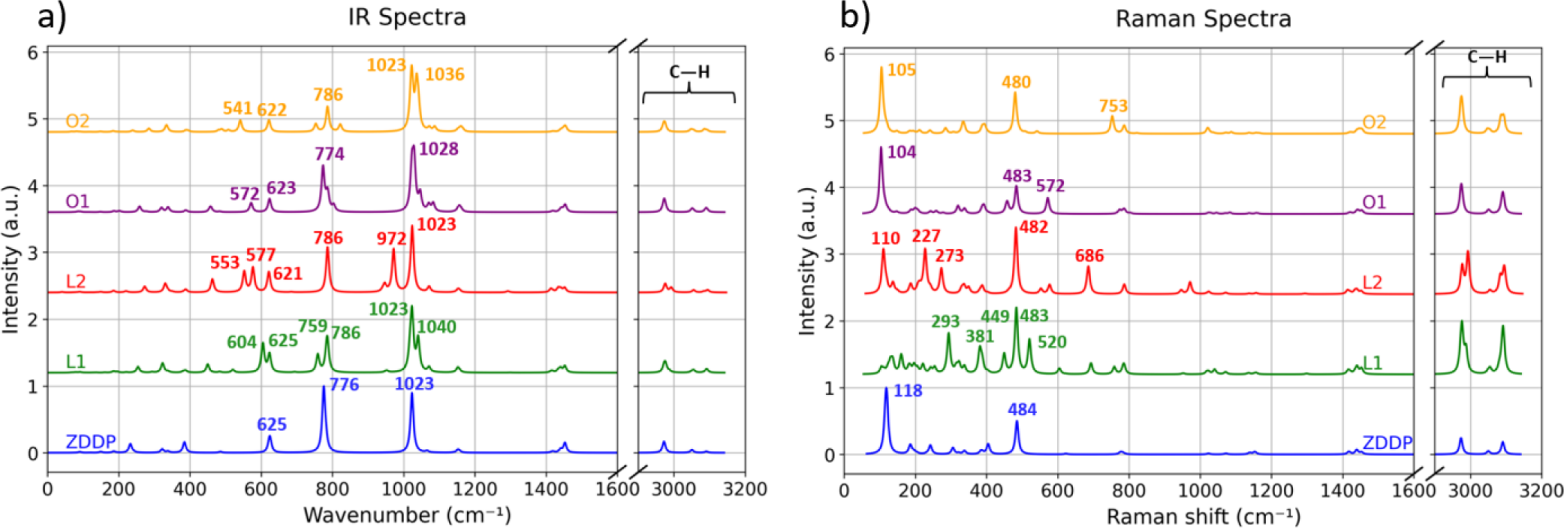
Calculated IR (a) and Raman (b) spectra for the investigated molecules. A vertical shift of 1.2 au is introduced to obtain a clear comparison. The colors indicate the considered molecules: blue is for standard ZDDP, green is for LI1-ZDDP, red is for LI2-ZDDP, purple is O1-ZDDP, and yellow is for O2-ZDDP, respectively.

Figure 3a shows the IR spectra of the investigated molecules. The spectral region between 1600 cm^–1^ and 2900 cm^–1^ was contracted, as no significant peaks were observed. Similarly, the peaks in the range of 2900 cm^–1^ to 3200 cm^–1^, corresponding to C–H vibrations in terminal methyl groups, are beyond the scope of this study. The most relevant features appear between 550 cm^–1^ and 1100 cm^–1^, where key vibrational modes are observed. The ZDDP spectrum (blue line) exhibits prominent peaks at 1023 cm^–1^ and 776 cm^–1^. The peak at 1023 cm^–1^ corresponds to C–O–P vibrations. This value is close to the experimental ones found in the literature, where the characteristic P–O–C peak region for ZDDP is found between 950 and 1020 cm^–1^.^59,60^ This mode is sensitive to molecular modifications: LI1-ZDDP, with one S-to-O swap, displays a shifted peak at 1040 cm^–1^, while LI2-ZDDP, with two S-to-O swaps, shows activity at 972 cm^–1^. These new peaks are attributed to P–O vibrations involving substituted oxygen atoms. In the oxidized derivatives O1-ZDDP and O2-ZDDP, additional oxygen atoms bonded to phosphorus broaden and alter the peak around 1023 cm^–1^, reflecting variations in the C–O–P vibration. The second characteristic peak for ZDDP, at 776 cm^–1^, corresponds to O–P–O vibrations. This peak also shifts in response to molecular modifications. For LI1-ZDDP, two peaks appear at 759 cm^–1^ and 786 cm^–1^, both associated with P–O vibrations. LI2-ZDDP displays a single shifted peak at 786 cm^–1^. In O1-ZDDP and O2-ZDDP, this region shows broader peaks with reduced intensity, attributed to O–P–O vibrations involving oxidized portions of the molecules. The peak at 625 cm^–1^ in ZDDP corresponds to S–P–S bond vibrations, in agreement with the literature.^61^ This mode is modified when S is substituted by O. For LI1-ZDDP, a new peak at 604 cm^–1^ reflects O–P–S interactions on the modified side of the molecule. In LI2-ZDDP, two peaks at 577 cm^–1^ and 553 cm^–1^ arise, corresponding to P–S and P–O vibrations, respectively. For O1-ZDDP and O2-ZDDP, peaks at 572 cm^–1^ and 541 cm^–1^ are observed, indicating O–P–O vibrations in the oxidized portions. Vibrational modes involving Zn, located in the lower wavenumber region, are less affected by molecular modifications.

The Raman spectra, calculated with an incident laser wavelength of 785 nm, are presented in Figure 3b. Molecular modifications significantly influence Raman activity, particularly in the range between 100 cm^–1^ and 800 cm^–1^. For ZDDP, two prominent peaks at 484 cm^–1^ and 118 cm^–1^ are observed. The peak at 484 cm^–1^ is attributed to S–P–S vibrations, while the peak at 118 cm^–1^ corresponds to the rotational modes of methyl groups around C–O bonds. In modified molecules, additional peaks populate this spectral region. In LI1-ZDDP, S-to-O swap introduces new peaks at 293 cm^–1^, 381 cm^–1^, 449 cm^–1^, and 520 cm^–1^, all corresponding to O–P–S vibrations on the modified side. LI2-ZDDP shows three new peaks: at 227 cm^–1^ due to S–P–S vibrations on the substituted side, at 273 cm^–1^ associated with vibrations of the central Zn atom, and at 686 cm^–1^ from C–S stretching. For O1-ZDDP, a new peak at 572 cm^–1^ is observed, corresponding to O–P–O vibrations in the oxidized portion of the molecule. Finally, O2-ZDDP exhibits a peak at 753 cm^–1^, attributed to O–P–O vibrations within the phosphate group. In situ Raman spectroscopy studies have reported the appearance of PO_3_ and PO_4_ bending vibration peaks at 525 and 440 cm^–1^, respectively. Additionally, a relatively broad P–O–P stretching vibration peak was observed in the 640–700 cm^–1^ range after 10 to 60 min of rubbing under boundary lubrication conditions.^62^ Assuming that oxidation of the molecule might occur during the initial stages of tribofilm formation, these spectral features may correspond to the emerging peaks we observe in the 400–750 cm^–1^ region for oxidized molecules. The calculated spectra provide a reference for further experimental studies to identify and evaluate the presence of ZDDP-modified molecules in tribological environments.

### Molecular Dissociation under 1 GPa Normal Load

After that, we aimed to investigate the effect of different coordination environments for the P atom on molecular dissociation on ferrous substrates. In fact, being the tribofilm composed of polyphosphates, the different stability of P bonds should affect significantly the dissociation of the molecule and the first steps of film formation. To do so, we compared uniaxial compression calculations under the effect of 1 GPa normal load of the five investigated molecules, namely ZDDP, LI1-ZDDP, LI2-ZDDP, O1-ZDDP and O2-ZDDP, both at Fe and 0.25 ML O-passivated Fe interfaces. This model has been already employed and tested by our group to efficiently describe the behavior of iron oxide for the study of molecular adsorption and dissociation of ZDDP.^36^ In that study, we also observed that the dissociation pathway of the molecule under applied shear stress—now recognized as a critical factor in tribofilm formation^14^—was analogous to that under normal load. In both cases, the dissociation led to the release of organophosphorus units, which serve as the fundamental building blocks of the tribofilm. The results of this step are reported in Figure 4a–d) (clean Fe) and in Figure 4f–j (0.25 ML O–Fe).

**Figure 4:**
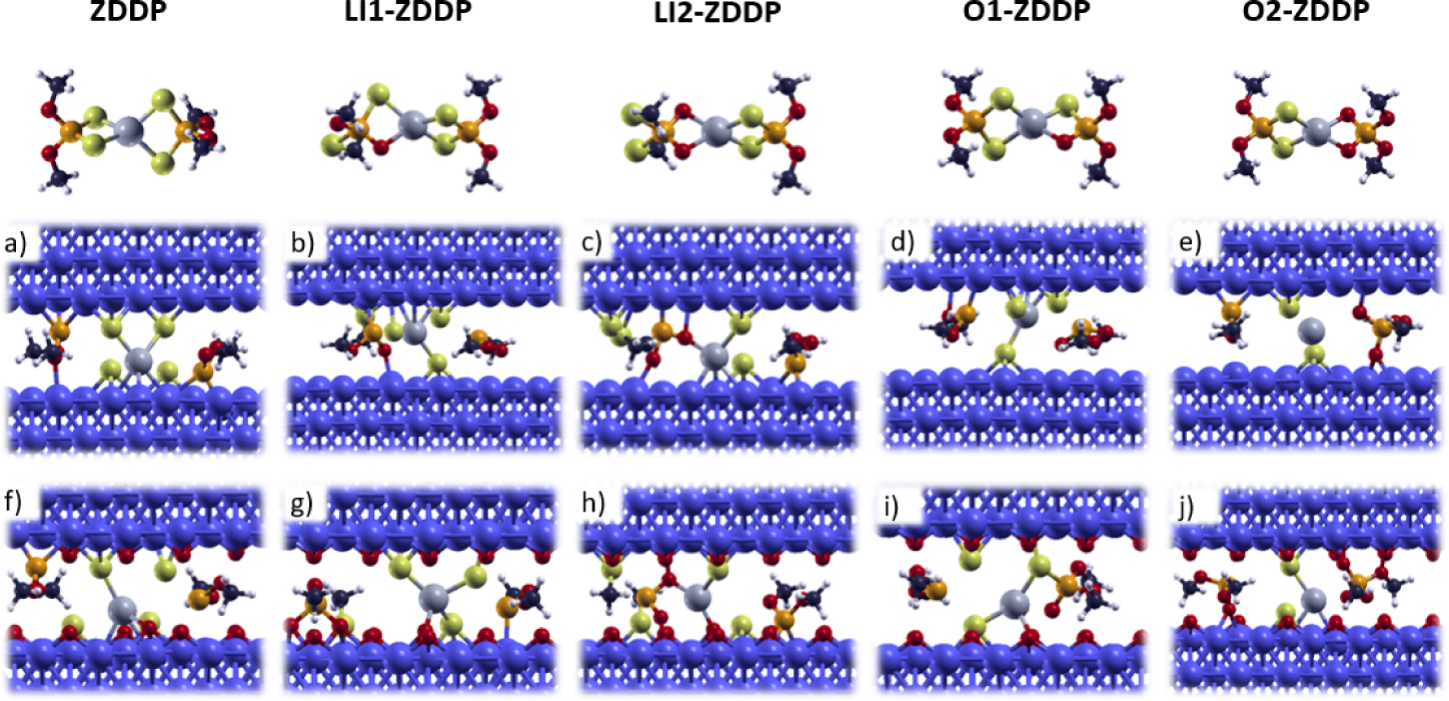
Final configurations obtained by vertical uniaxial compressions in which a normal 1 GPa load is applied to a cell containing the molecule on a Fe (a–e) and 0.25 ML O-passivated Fe (f–j) slab. On top, the five investigated molecules: standard ZDDP, LI1-ZDDP, LI2-ZDDP, O1-ZDDP, and O2-ZDDP.

These simulations show that, in general, the primary dissociation separates the central Zn–S unit from the two lateral P-based units. The Zn–S unit instantly chemisorb to the substrate. Concerning the P-based one, it is worth noting that S–P bonds are among the first to break, followed by the strong chemisorption of atomic S and organophosphorus units separately. Interestingly, P–O bonds seem to be the hardest to break: in fact, O1-ZDDP and O2-ZDDP (Figure 4d,i and Figure 4e,j, respectively) contain phosphite and phosphate units which are preserved and do not dissociate. At the same time, LI1-ZDDP and LI2-ZDDP (Figure 4b,g and Figure 4c,h, respectively) which are characterized by S-to-O swaps, lose the S-chain units, while the P–O bonds are preserved.

### Bonds Strength Analysis: Static Fragmentation and ICOOP

To quantify the relative strength of these bonds, we performed static fragmentations of the molecules by computing the total energies of the fragments obtained by four different cuts, displayed in Figure 5, and comparing them with that of the full molecule. These energy differences, reported in the picture, represent the energy required to break the bonds corresponding to each cut. Additionally, Integrated Crystal Orbital Overlap Population (ICOOP) analysis was performed to gain insight into the electronic contributions to bond strengths.

**Figure 5:**
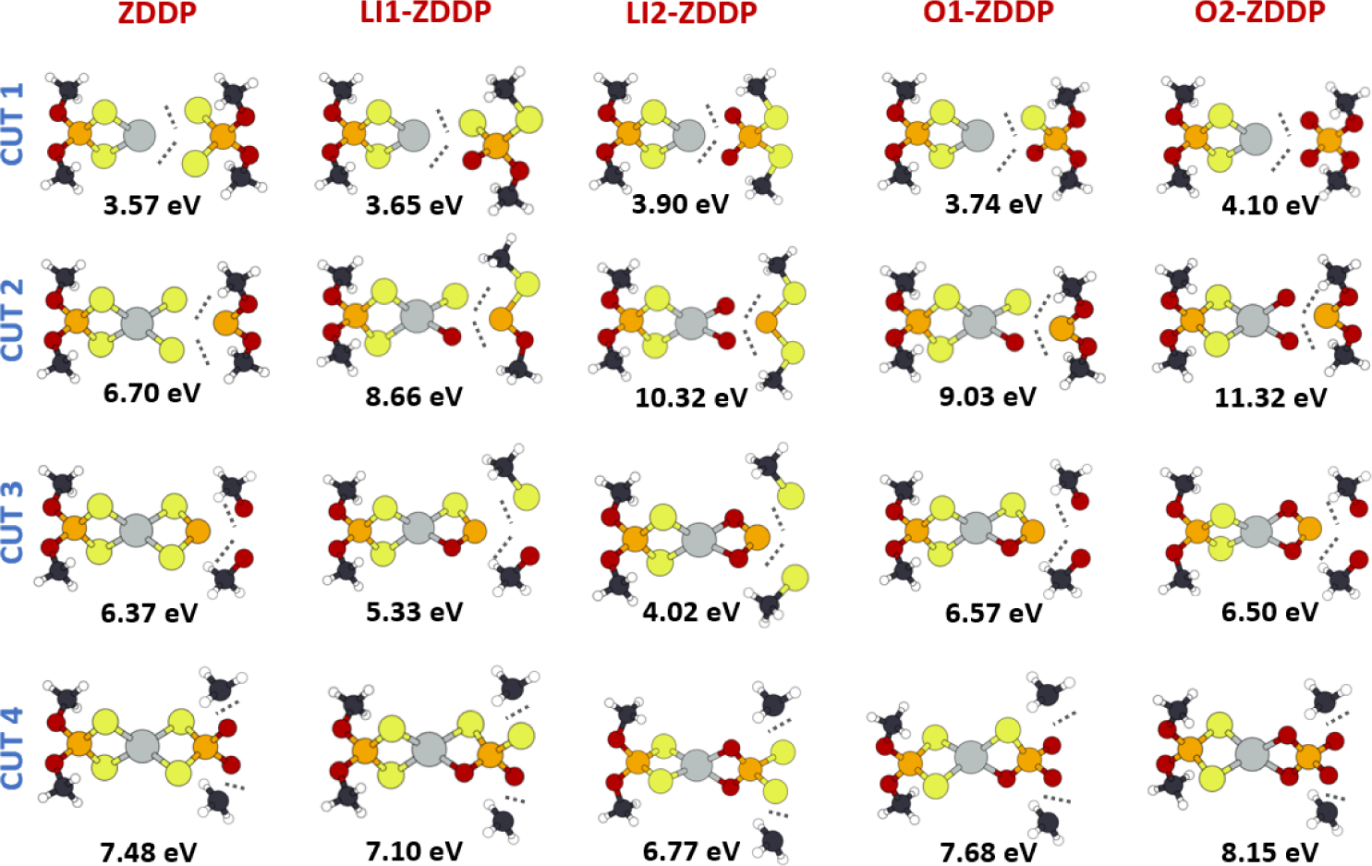
Four investigated cuts (rows) of the five investigated molecules (columns). The fragmentation energies *E*_frag_ are reported for each investigated configuration.

The first row in Figure 5 summarizes the results for cut 1, involving the cleavage of two Zn–S or Zn–O bonds. In ZDDP, this fragmentation requires 3.57 eV, corresponding to the breaking of two Zn–S bonds. In contrast, LI2-ZDDP and O2-ZDDP, which involve the cleavage of two Zn–O bonds, require higher fragmentation energies of 3.90 and 4.10 eV, respectively. Molecules such as LI1-ZDDP and O1-ZDDP, where the cut severs one Zn–S and one Zn–O bond, exhibit intermediate fragmentation energies of 3.65 and 3.74 eV, respectively. The higher energy cost associated with the breaking of Zn–O bonds compared to Zn–S provides an explanation to the higher stability of linkage isomers compared to the standard molecule, presented in the “ZDDP Linkage Isomers” Section. The trend in fragmentation energies is reflected in the ICOOP values. Zn–O bonds in O2-ZDDP and LI2-ZDDP have higher ICOOP values (0.020–0.026 and 0.025, respectively) compared to those in O1-ZDDP and LI1-ZDDP (0.017 and 0.018). This indicates stronger Zn–O bonds in O2-ZDDP and LI2-ZDDP. Similarly, the Zn–S bonds in ZDDP, with an ICOOP value of 0.066, are weaker than those in LI1-ZDDP (0.088) and O1-ZDDP (0.086), correlating with the lower fragmentation energy for ZDDP. Overall, Zn–O bonds are stronger in the oxidized derivatives, while Zn–S bonds are slightly stronger in molecules where they coexist with Zn–O bonds. The second row of Figure 5 details the fragmentation energies for cut 2, which involves breaking P–O and P–S bonds. The highest energy required to sever two P–O bonds is for LI2-ZDDP and O2-ZDDP (10.32 and 11.32 eV, respectively). ZDDP, which involves the cleavage of two P–S bonds, requires only 6.70 eV, the lowest energy among the molecules. LI1-ZDDP and O1-ZDDP, where the cut involves one P–O bond and one P–S bond, have intermediate fragmentation energies of 8.66 and 9.03 eV, respectively. The ICOOP analysis explains these trends. P–O bonds in oxidized derivatives exhibit higher ICOOP values, indicating greater bond strength. Indeed, LI1-ZDDP has an ICOOP value for P–O of 0.275, whereas O1-ZDDP has a value of 0.307. Analogously, P–O in LI2-ZDDP has an ICOOP value of 0.289, to be compared with that of O2-ZDDP, which is 0.339. In contrast, P–S bonds exhibit smaller and relatively uniform ICOOP values across all molecules, confirming their weaker and less variable nature. Consequently, the stability of phosphate and phosphite groups significantly increases the energy required for fragmentation. The third row of Figure 5 presents fragmentation energies for cut 3, which involves the detachment of methyl terminations attached to oxygen or sulfur. Molecules with P–S bond cleavage, such as LI1-ZDDP and LI2-ZDDP, require lower fragmentation energies (5.33 and 4.02 eV, respectively). By contrast, cleavage of alkoxy chains, as in O1-ZDDP and O2-ZDDP, is more energetically demanding. The highest energy is observed for O1-ZDDP (6.57 eV), followed by O2-ZDDP (6.50 eV). The ICOOP values support this trend. For molecules involving the cleavage of P–O bonds, such as O1-ZDDP and O2-ZDDP, the ICOOP values are the highest (0.211 for O1-ZDDP and 0.250 for O2-ZDDP), reflecting the strong P–O bonds in these derivatives. ZDDP, with the lowest ICOOP value for P–O bonds (0.175), exhibits a correspondingly lower fragmentation energy (6.37 eV). The fourth row of Figure 5 describes cut 4, which involves the cleavage of alkyl chains from oxygen or sulfur atoms. The fragmentation energy is higher for cuts involving O–C bonds compared to those involving S–C bonds. For instance, LI2-ZDDP, which involves the breaking of two S–C bonds, has the lowest fragmentation energy (6.77 eV). In contrast, molecules with O–C bond cleavage, such as O1-ZDDP and O2-ZDDP, require higher fragmentation energies (7.68 and 8.15 eV, respectively). Interestingly, the ICOOP values for O–C bonds do not vary significantly among the investigated molecules. In general, cut 1 is the less energetically expensive for all the investigated molecules, followed by cut 3. Except for ZDDP, cut 2 is the most energetically demanding: this is due to the breaking of the strong P–O bonds in the alternative forms of the molecule.

## Conclusions

This study provides a comprehensive computational analysis of zinc dialkyldithiophosphate (ZDDP) and its isomers, with a focus on molecular stability, oxidative degradation, vibrational spectra, dissociation behavior under tribological conditions, and bond strength. The key findings can be summarized as follows:

- The optimization of ZDDP linkage isomers revealed that S-to-O swap consistently stabilize the molecule relative to the standard ZDDP structure, in agreement with the existing literature.^25^ This can be explained by the higher strength of Zn–O bonds compared to Zn–S ones.
- By presenting the first stability diagram for this phenomenon, we provide direct evidence that ZDDP undergoes energetically favorable oxidative degradation either in the presence of O_2_ or via substrate-mediated pathways. This novel insight aligns with previous observations for MoDTC,^28^ as well as experimental studies reporting S-to-O substitutions in oxygen-rich environments during ZDDP oxidation.^49,50^

- Vibrational spectra calculations (IR and Raman) revealed significant shifts and changes in peak intensities corresponding to S-to-O swaps and oxidative modifications. Key vibrational modes, such as those involving P–O and P–S bonds, exhibited predictable patterns linked to molecular modifications, offering potential markers for further experimental characterization. These results relate closely to experimental evidence from in situ Raman spectroscopy, where the emergence of PO_3_ and PO_4_ bending vibrations, along with a broad P–O–P stretching peak during the early stages of tribofilm formation, has been reported.^62^ This observation may be attributed to oxidation of the molecule.
- Simulations of ZDDP dissociation under 1 GPa load indicated that molecular breakdown initiates with the separation of the Zn–S unit, followed by the fragmentation of S–P bonds. P–O bonds, particularly in oxidized derivatives (O1-ZDDP and O2-ZDDP), were notably more resistant to dissociation, emphasizing their potential role in stabilizing polyphosphate structures in tribofilms. These results suggest that molecular oxidation significantly influences ZDDP dissociation and, consequently, the kinetics of tribofilm formation. This may help explain experimentally observed differences in tribofilm durability and formation rates under oxygen-rich conditions compared to inert atmospheres.^19^
- Fragmentation energy calculations and Integrated Crystal Orbital Overlap Population (ICOOP) analyses highlighted the enhanced strength of Zn–O and P–O bonds in oxidized molecules compared to Zn–S and P–S bonds in the standard ZDDP. The results provide quantitative evidence of the increased stability introduced by S-to-O swaps.

Overall, these findings advance our understanding of ZDDP behavior at the molecular level, providing valuable insights into its tribological performance and degradation mechanisms. The results underscore the significance of oxidative processes in tribofilm formation and offer a foundation for the rational design of more efficient lubricant additives. In this context, the static calculations presented here elucidate the atomic-scale mechanisms of ZDDP dissociation, offering direct evidence of how chemical modifications influence its reactivity. Specifically, our findings demonstrate that isomerization and oxidation are pivotal in affecting the initial molecular dissociation processes on the substrate that precede tribofilm formation, by markedly enhancing P–O bond strength and enabling the formation of highly stable organophosphorus units.

We successfully applied this approach in previous works to study tribological systems.^35,63,64^ These static insights form a critical basis for future molecular dynamics simulations, which will enable a more comprehensive assessment of the tribological behavior of modified ZDDP molecules under dynamic conditions.
